# Temporal and spatial trends in suicide-related visits before and during the COVID-19 pandemic in the US, 2018–2021

**DOI:** 10.1016/j.jad.2022.12.062

**Published:** 2023-03-01

**Authors:** Yunyu Xiao, Alvin Junus, Tianzi Li, Paul Yip

**Affiliations:** a Department of Population Health Sciences, Weill Cornell Medicine, NewYork-Presbyterian, New York, United States; b Department of Social Work and Social Administration, The University of Hong Kong, Hong Kong; c HKJC Centre for Suicide Research and Prevention, The University of Hong Kong, Hong Kong

**Keywords:** Suicide attempts, Suicidal ideation, Electronic health records, COVID-19

## Abstract

**Background:**

COVID-19 disproportionately impacted mental health in disadvantaged populations and areas. However, disparities in long-term changes in suicide-related visits across the US are unclear. This retrospective study aims to characterize temporal and spatial changes in suicide-related visits in healthcare settings from 2018 to 2021 in the U.S.

**Methods:**

We use electronic health records for 21,860,370 patients from Healthjump through the COVID-19 Research Database Consortium. Healthjump harmonizes EHR data from over 55 national databases across the US. Suicide ideation and suicide attempts between January 1, 2018 and December 12, 2021 were identified by the diagnosis codes in 6 periods in 2021 compared with the same periods in 2018–2020.

**Results:**

There was 30,019 suicidal ideation, and 7392 suicide attempt visits from January 2018 to November 2021. 15–20-year-olds were the most represented age group at 6302 suicide ideation visits (21.0 % of suicide ideation visits) and 1326 suicide attempt visits (17.9 % of suicide attempt visits), followed by suicide-related visits among 60+ years old. Compared with pre-pandemic periods, youth aged 15–20, females, White, non-Hispanic, and English speakers had increased suicide-related visits, especially suicidal ideation (*P* < 0.05). Suicide attempts with non-medical substances increased to 28.0 % in the first 6 months of the pandemic in 2020, compared with the prior year (21.5 %). COVID-19 patients had increased suicidal ideation in 2020.

**Limitations:**

The EHR data is not nationally representative.

**Conclusions:**

This study found significant and disproportionate increases in suicide related visits over the COVID-19 stages. To prevent the next storms of suicides, future interventions shall accommodate needs among vulnerable groups during and after periods of crisis.

## Highlights

• This study analyzes temporal and spatial changes in suicide-related visits in healthcare settings from 2018-2021 in U.S.
• Females, youth aged 15-20, White, non-Hispanic, and English speakers had increased post-pandemic suicide-related visits.
• Suicide attempts with non-medical substances increased from 21.5% to 28.0% in late 2021.
• COVID-19 patients had increased suicidal ideation in 2020.
• Suicide-related visits among patients aged 50-59 years old decreased after the vaccination rollout.

## 1 Introduction

The coronavirus disease 2019 (COVID-19) pandemic has raised an emerging concern about mental health and suicide risks globally and nationally (Holland et al., 2021; Ridout et al., 2021; Xiao et al., 2022b). While earlier data indicate no significant increase in suicide death rates (John et al., 2020; Pirkis et al., 2021), there are concerning increases in suicide attempts based on evidence from the early pandemic (mid-March through October 2020), compared with the same period in 2019 (Cousien et al., 2021; Ridout et al., 2021). Importantly, there has been a desperate mental health impact of COVID-19 (Czeisler et al., 2020; Penninx et al., 2022; Xiao et al., 2022a). In addition to disproportionate exposures to COVID-19 infections, there are mental health disparities associated with changes in social determinants of health (SDoH) impacted by COVID-19, including job loss, barriers to health care, school disruptions, and housing insecurity (Czeisler et al., 2021; Reger et al., 2020; Xiao et al., 2022a; Yip et al., 2010; Yip and Chau, 2020). Pre-existing mental health disparities have disproportionately worsened mental health and increased suicide risks among vulnerable groups, including females, youth, and communities of colors (Hill et al., 2021; Ridout et al., 2021; Spencer et al., 2021; Xiao et al., 2022a). As SDoH constantly drives potential health disparities, it is essential to monitor and address the disparate impact of COVID-19 on suicidal ideation and attempts in the longer term.

However, reliable empirical evidence delineating the heterogeneous trends of suicidal ideation and suicide attempts at the national level before COVID-19 and during different stages of COVID-19 in the long term remains scant. The majority of the existing studies are based on aggregated suicide statistics (Curtin et al., 2021), proxies of suicide-related behaviors such as Google health trends (Halford et al., 2020; Manzar et al., 2021), small samples or convenient sampling from online surveys (Czeisler et al., 2020; Isumi et al., 2020; Ueda et al., 2021). Existing literature is limited by the lack of pre-COVID-19 comparisons and short-term investigations within one year of the pandemic (Penninx et al., 2022). Few examined spatial differences across the US despite the high variations in government responses by states (Hale, 2022).

Inclusive assessment of suicide trends requires harmonized data covering near-real-time, long-term, nationwide, and large samples at the disaggregated level and information from both pre-COVID-19 periods (to serve as the baseline) and post-COVID-19 periods. Policymakers also need important evidence that disentangles any disparities in trends of suicidal ideation and suicide attempts by demographic characteristics, geographic locations, and COVID-19 status, as observed in prior studies on other health outcomes (Gu et al., 2020; Hooper et al., 2020; Khatri et al., 2021; Kim et al., 2020; Lopez et al., 2021; Muñoz-Price et al., 2020).

Using data from a large, electronic health record (EHR) integrated system across the US, we compared trends in suicide-related visits (suicidal ideation, suicide attempts) in the office, emergency departments, inpatient hospitals, and ambulatory surgical centers in all ages from January 1, 2018, to November 30, 2021. We examined differential trends in suicidal ideation and suicide attempt visits at six stages of the pandemic during COVID-19. To address the inequalities associated with SDoH in trends of suicide-related visits across different stages of the COVID-19 pandemic, we conducted subgroup analyses by sex, age group, race, ethnicity, preferred language, and geographic location. We further analyzed the trends in suicide methods, COVID-19 infection, and COVID-19 vaccination status.

## 2 Methods

### 2.1 Study design, setting, and population

This descriptive time series analysis used the Healthjump database (Healthjump Inc., Philadelphia, PA, USA) from the COVID-19 Research Database Consortium (https://covid19researchdatabase.org) funded by the Bill and Melinda Foundation. There were 21,860,370 patients during our study period from January 2018 to November 30, 2021. The COVID-19 research database (CRD) contains electronic health records (EHR), claims, and other consumer data from surveys. The database is curated to conduct real-world data studies related to COVID-19. Healthjump is a data management platform that solves interoperability challenges by integrating and harmonizing over 55 EHR and practice management systems (e.g., Athenahealth, Cerner, Epic, NextGen). Therefore, it is diverse and more representative of the US population. Healthjump database contains EHR across the US. The number of patient charts been increasing from 2018 (10,737,790), 2019 (12,566,233), 2020 (14,687,568), to 2021 (18,100,731). Healthjump EHR data includes diagnosis, procedures, labs, vitals, medications, and histories sourced from participating members of the Healthjump network. All personal information in this database was provided anonymously. Data were extracted by SQL in Snowflake (Snowflake Inc., San Mateo, CA, USA).

The advantage of Healthjump data is the rapid accessibility (data is refreshed daily) for a large US population. The majority of patients in Healthjump come from the southern region of the US (38,343,198 [44.05 %]), followed by the western region (20,581,887 [23.65 %]), Mideastern region (16,853,012 [19.36 %]), Northeastern region (11,247,994 [12.92 %]) and other regions (12,524 [0.01 %]). Most encounters (78.4 %) were office-based, followed by 4.54 % in the inpatient hospital, 2.36 % in the ambulatory surgical center, and 1.48 % from on-campus inpatient hospitals. Healthjump databases update when connecting to a new data source, such as a new EHR, a provider with a new specialty, or new visit types. Therefore, the dynamic nature of the data set provides a unique opportunity to offer a real-world reflection of suicidal ideation and suicide attempt visits but also prevents creating a snapshot of the data as the sample grows daily. Few changes in the geographic distributions of healthcare facilities contributed to the suicide-related visits in the Healthjump database over the study periods between 2018 and 2021 (eFig. 1). The Weill Cornell Medicine institutional review board deemed this analysis exempt from review, and informed consent was not required (WCM IRB#21–12,024,247). This study followed the Strengthening the Reporting of Observational Studies in Epidemiology (STROBE) reporting guideline.

### 2.2 Measures

*Suicide-related encounters* were identified by the ninth and tenth revisions of the International Statistical Classification of Diseases, Clinical Modification (ICD-9-CM, ICD-10-CM) diagnostic codes, and clinical findings under SNOMED Clinical Terms (SNOMED CT). These encounters constituted codes for suicidal ideation (ICD codes R45.851, V62.84, and SNOMED code 6471006) and different methods of suicide attempts (eMethod 1).

*Demographic characteristics* included patient-reported sex, age group, race, ethnicity, and preferred language. We further investigated spatial variations by states.

*COVID-19 characteristics* included the identification of patients who had tested positive for COVID-19 (using ICD-10 codes U07.1, U07.2) and those who had been vaccinated using records with a CVX code of 207 (Moderna), 208 (Pfizer), 210 (Oxford-AstraZeneca), 212 (Janssen), or CPT codes of 91300 (Pfizer), 91301 (Moderna), 91302 (Oxford-AstraZeneca), 91303 (Janssen).

Time and event variables included six different time intervals (eTable 1) corresponding to the pre-COVID-19 and pre-first case (01/01/2018–11/12/2019), pre–COVID-19 pandemic period (12/12/2019–29/02/2020), post–COVID-19 and first wave (01/03/2020–31/08/2020), post–COVID-19, post-first wave, and pre-vaccination (01/09/2020–12/12/2020), post–COVID-19, post-vaccination (pre-Delta) (13/12/2020–26/07/2021) and post–COVID-19, Delta wave (27/07/2021–30/11/2021). We chose July 27, 2021, as the cutoff as the Delta variant (B.1.617.2) surged during the summer of 2021 based on the CDC monitoring of variant proportions (CDC, 2022a) and the CDC definitions of different stages of COVID-19 infections (Johnson, 2022). On July 27, the CDC also recommended that all persons, including those who are fully vaccinated, should wear masks in indoor public settings in high COVID-19 transmission areas (CDC, 2022b). Our date cutoffs were consistent with the previous literature studying changes in COVID-19 infections and deaths by types of variants (Chun et al., 2022; Martin et al., 2022).

### 2.3 Statistical analysis

We calculated the frequencies and proportions of suicide-related visits by demographic characteristics, suicide attempt methods, and COVID-19 characteristics. For example, the proportion of males in suicidal ideation visits in the pre-COVID-19 period was calculated by dividing the number of male patients for suicidal ideation visits in the pre-COVID-19 period by the total number of patients for suicidal visits in the pre-COVID-19 period. Data were examined and compared across time through pairwise proportionality tests, with *P*-values adjusted by the Benjamini-Hochberg method to allow for comparisons of multiple groupings. All statistical tests were 2-sided, with an adjusted P-value ≤ 0.05 considered significant. All analyses were conducted with R version 4.1.2.

## 3 Results

### 3.1 Temporal and spatial changes in suicide-related encounters before and during the COVID-19 pandemic

Table 1 presents the descriptive statistics among the study sample, including 30,019 suicidal ideation and 7392 suicide attempt visits. From January 2018 to November 2021, the frequency of suicidal ideation visits increased by 84.0 % (frequency ratio was 1.8), reaching the peak twice (one in October 2020, the other in April 2021). The frequency of suicide attempt visits increased by 115.0 % (frequency ratio was 2.2) and peaked in October 2021 (see eTable 2 and eFigure 2 for the monthly frequency and percentages of suicidal ideation and suicide attempts). 17,990 (59.9 %) of suicide ideation visits and 4377 (59.2 %) suicide attempt visits were female. Patients aged 15–20-year-olds were the most represented age group at 6302 suicide ideation visits (21.0 % of suicide ideation visits) and 1326 suicide attempt visits (17.9 % of suicide attempt visits), followed by patients over 60 years old, who comprised 3837 suicide ideation visits (12.8 % of suicide ideation visits) and 1222 suicide attempt visits (16.5 % of suicide attempt visits). Most suicide-related visits involved people who were White (15,235 [50.8 %] of suicide ideation visits and 4195 [56.8 %] of suicide attempt visits), non-Hispanic (17,558 [58.5 %] of suicide ideation visits and 4257 [57.6 %] of suicide attempt visits), and who were English speakers (18,681 [62.2 %] of suicide ideation visits and 4533 [61.3 %] of suicide attempt visits). Since the first case of COVID-19 on December 2, 2019, among patients who tested positive for COVID-19. There has been increasing suicidal ideation from 115 (6.3 %) in the pre-pandemic period to the first wave of the pandemic (254 [7.8 %]) and the Delta wave (320 [9.2 %]); and suicide attempts from 21 (5.4 %) in the pre-pandemic period to 49 (9.1 %) after the first-wave since the pandemic, which slightly decreased to 71 (8.1 %) in the Delta wave.

**Table 1:** Descriptive characteristics of suicide-related visits in six distinct time intervals from 01/01/2018 to 30/11/2021.

Characteristics	N. (%)						
		Pre-COVID-19		Post-COVID-19			
	Total	Pre first case	Pre-pandemic	First wave	Post-first wave, pre-vaccination	Post-vaccination, pre-delta	Delta wave
	01/01/2018–30/11/2021	01/01/2018–11/12/2019	12/12/2019–29/02/2020	01/03/2020–31/08/2020	01/09/2020–12/12/2020	13/12/2020–26/07/2021	27/07/2021–30/11/2021
Suicidal ideation	*n* = *30,019*	*n* = *12,851*	*n* = *1842*	*n* = *3252*	*n* = *2581*	*n* = *6004*	*n* = *3489*

Sex							
Male	12,026 (40.1 %)	5347 (41.6 %)	766 (41.6 %)	1305 (40.1 %)	946 (36.7 %)	2323 (38.7 %)	1339 (38.4 %)
Female	17,990 (59.9 %)	7504 (58.4 %)	1075 (58.4 %)	1947 (59.9 %)	1635 (63.4 %)	3680 (61.3 %)	2149 (61.6 %)
Unknown	3 (0.0 %)	0 (0.0 %)	1 (0.1 %)	0 (0.0 %)	0 (0.0 %)	1 (0.0 %)	1 (0.0 %)

Age (years)							
<15	3684 (12.3 %)	1378 (10.7 %)	239 (12.0 %)	314 (9.7 %)	380 (14.7 %)	827 (13.8 %)	546 (15.7 %)
15–20	6302 (21.0 %)	2443 (19.0 %)	434 (23.6 %)	680 (20.9 %)	557 (21.6 %)	1392 (23.2 %)	796 (22.8 %)
21–25	3373 (11.2 %)	1401 (10.9 %)	213 (11.6 %)	389 (12.0 %)	315 (12.2 %)	690 (11.5 %)	365 (10.5 %)
26–30	2553 (8.5 %)	1183 (9.2 %)	103 (5.6 %)	301 (9.3 %)	213 (8.3 %)	479 (8.0 %)	274 (7.9 %)
31–39	3753 (12.5 %)	1636 (12.7 %)	200 (10.9 %)	521 (16.0 %)	301 (11.7 %)	710 (11.8 %)	385 (11.0 %)
40–49	3382 (11.3 %)	1572 (12.2 %)	224 (12.2 %)	356 (11.0 %)	230 (8.9 %)	619 (10.3 %)	381 (10.9 %)
50–59	3135 (10.4 %)	1534 (11.9 %)	172 (9.3 %)	342 (10.5 %)	242 (9.4 %)	527 (8.8 %)	318 (9.1 %)
60+	3837 (12.8 %)	1704 (13.3 %)	257 (14.0 %)	349 (9.7 %)	343 (13.3 %)	760 (12.7 %)	424 (12.2 %)

Race							
White	15,235 (50.8 %)	6065 (47.2 %)	990 (53.8 %)	1675 (51.5 %)	1395 (54.1 %)	3165 (52.7 %)	1945 (55.8 %)
Non-white	2716 (9.1 %)	1119 (8,7 %)	165 (9.0 %)	296 (9.1 %)	240 (9.3 %)	589 (9.8 %)	307 (8.8 %)
Unknown	12,068 (40.2 %)	5667 (44.1 %)	687 (37.3 %)	1281 (39.4 %)	946 (36.7 %)	2250 (37.5 %)	1237 (35.5 %)

Ethnicity							
Hispanic	3770 (12.6 %)	1619 (12.6 %)	230 (12.5 %)	403 (12.4 %)	284 (11.0 %)	791 (13.2 %)	443 (12.7 %)
Non-Hispanic	17,558 (58.5 %)	7174 (55.8 %)	1100 (59.7 %)	1964 (60.4 %)	1545 (59.9 %)	3578 (59.6 %)	2197 (63.0 %)
Unknown	8691 (29.0 %)	4058 (31.6 %)	512 (27.8 %)	885 (27.2 %)	752 (29.1 %)	1635 (27.2 %)	849 (24.3 %)

Primary language							
English	18,681 (62.2 %)	7542 (58.7 %)	1125 (61.1 %)	2062 (63.4 %)	1622 (62.8 %)	3955 (65.9 %)	2375 (68.1 %)
Non-English	4370 (14.6 %)	1865 (14.5 %)	311 (16.9 %)	493 (15.2 %)	359 (13.9 %)	832 (13.9 %)	510 (14.6 %)
Unknown	6968 (23.2 %)	3444 (26.8 %)	406 (22.0 %)	697 (21.4 %)	600 (23.3 %)	1217 (20.3 %)	604 (17.3 %)
COVID-19 positive	1453 (8.5 %)	–	115 (6.3 %)	254 (7.8 %)	231 (9.0 %)	533 (8.9 %)	320 (9.2 %)
Vaccinated	355 (3.7 %)	–	–	–	–	216 (3.6 %)	139 (4.0 %)

Suicide attempt	*n* = *7392*	*n* = *3373*	*n* = *396*	*n* = *854*	*n* = *537*	*n* = *1357*	*n* = *875*

Sex							
Male	3011 (40.7 %)	1408 (41.7 %)	173 (43.7 %)	346 (40.5 %)	214 (39.9 %)	533 (39.3 %)	337 (38.5 %)
Female	4377 (59.2 %)	1964 (58.2 %)	222 (56.1 %)	508 (59.5 %)	323 (60.2 %)	823 (60.7 %)	537 (61.4 %)
Unknown	4 (0.1 %)	1 (0.0 %)	1 (0.3 %)	0 (0.0 %)	0 (0.0 %)	1 (0.1 %)	1 (0.1 %)

Age (years)							
<15	864 (11.7 %)	331 (9.8 %)	32 (8.1 %)	101 (11.8 %)	75 (14.0 %)	195 (14.4 %)	130 (14.9 %)
15–20	1326 (17.9 %)	562 (16.7 %)	84 (21.2 %)	146 (17.1 %)	94 (17.5 %)	272 (20.0 %)	168 (19.2 %)
21–25	756 (10.2 %)	312 (9.3 %)	55 (13.9 %)	87 (10.2 %)	71 (13.2 %)	135 (10.0 %)	96 (11.0 %)
26–30	479 (6.5 %)	235 (7.0 %)	29 (7.3 %)	50 (5.9 %)	24 (4.5 %)	80 (5.9 %)	61 (7.0 %)
31–39	880 (11.9 %)	435 (12.9 %)	42 (10.6 %)	107 (12.5 %)	66 (12.3 %)	149 (11.0 %)	81 (9.3 %)
40–49	928 (12.6 %)	453 (13.4 %)	41 (10.4 %)	114 (13.4 %)	64 (11.9 %)	170 (12.5 %)	86 (9.8 %)
50–59	937 (12.7 %)	490 (14.5 %)	46 (11.6 %)	119 (13.9 %)	61 (11.4 %)	135 (10.0 %)	86 (9.8 %)
60+	1222 (16.5 %)	555 (16.5 %)	67 (16.9 %)	130 (15.2 %)	82 (15.3 %)	221 (16.3 %)	167 (19.1 %)

Race							
White	4195 (56.8 %)	1868 (55.4 %)	212 (53.5 %)	493 (57.7 %)	297 (55.3 %)	794 (58.5 %)	531 (60.7 %)
Non-white	653 (8.8 %)	283 (8.4 %)	47 (11.9 %)	87 (10.2 %)	49 (9.1 %)	110 (8.1 %)	77 (8.8 %)
Unknown	2544 (34.4 %)	1222 (36.2 %)	137 (34.6 %)	274 (32.1 %)	191 (35.6 %)	453 (33.4 %)	267 (30.5 %)

Ethnicity							
Hispanic	989 (13.4 %)	447 (13.3 %)	60 (15.2 %)	127 (14.9 %)	55 (10.2 %)	184 (13.6 %)	116 (13.3 %)
Non-Hispanic	4257 (57.6 %)	1875 (55.6 %)	2219 (55.3 %)	479 (56.1 %)	308 (57.4 %)	825 (60.8 %)	551 (63.0 %)
Unknown	2146 (29.0 %)	1051 (31.2 %)	117 (29.6 %)	248 (29.0 %)	174 (32.4 %)	348 (25.6 %)	208 (23.8 %)

Primary language							
English	4533 (61.3 %)	1921 (57.0 %)	247 (62.4 %)	507 (59.4 %)	339 (63.1 %)	900 (66.3 %)	619 (70.7 %)
Non-English	1247 (16.9 %)	609 (18.1 %)	61 (15.4 %)	156 (18.3 %)	68 (12.7 %)	221 (16.3 %)	132 (15.1 %)
Unknown	1612 (21.8 %)	843 (25.0 %)	88 (22.2 %)	191 (22.4 %)	130 (24.2 %)	236 (17.4 %)	124 (14.2 %)

Methods of suicide							
Firearms	476 (6.4 %)	201 (6.0 %)	29 (7.3 %)	73 (8.6 %)	55 (10.2 %)	76 (5.6 %)	42 (4.8 %)
Poisoning - drugs	3109 (42.1 %)	1484 (44 %)	163 (41.2 %)	324 (37.9 %)	232 (43.2 %)	551 (40.6 %)	355 (40.6 %)
Poisoning - other toxic substances	1748 (23.7 %)	832 (24.7 %)	87 (22.0 %)	239 (28.0 %)	97 (18.1 %)	281 (20.7 %)	212 (24.2 %)
Other methods	2059 (27.9 %)	856 (25.4 %)	117 (29.6 %)	218 (25.5 %)	153 (28.5 %)	449 (33.1 %)	266 (30.4 %)
COVID-19 positive	315 (7.8 %)		21 (5.4 %)	65 (7.6 %)	49 (9.1 %)	109 (8.0 %)	71 (8.1 %)
Vaccinated	42 (1.9 %)					20 (1.5 %)	22 (2.5 %)

*Note*. Percentage points are rounded to one decimal place, and thereby some proportions may not add up to exactly 100.

**Fig. 1:**
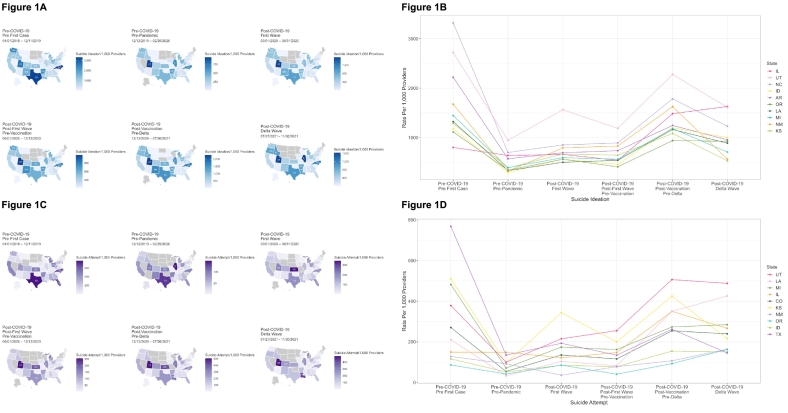
Temporal and Spatial changes in suicidal ideation and suicide attempt visits, 2018–2021. *Note*. Rates were calculated by the frequency of suicidal ideation and suicide attempts per 1000 healthcare providers to adjust for the changing number of providers over time.

Fig. 1 presents the spatial variations in suicide-related visits before and during COVID-19 across all states with available data from 2018 to 2021. Utah, North Carolina, Illinois, and Texas were among the states with consistently high suicidal ideation (Fig. 1A) and suicide attempts (Fig. 1C). Suicidal ideation rates increased the most in Illinois (Fig. 1B), while suicide attempts increased the most in Utah and Louisiana during COVID-19 (Fig. 1D), compared to the previous study periods.

### 3.2 Temporal differences in suicide-related visits by sex, age group, race, ethnicity, and preferred language

**Fig. 2:**
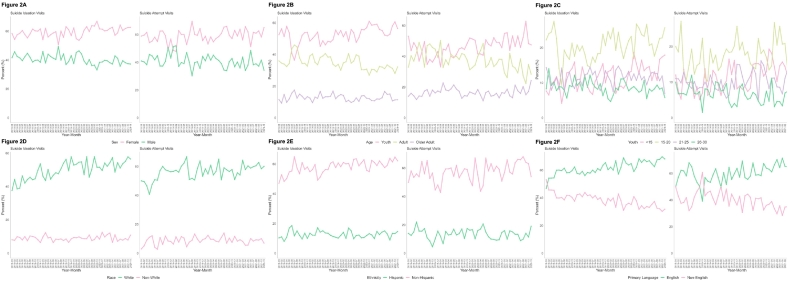
Temporal changes of suicidal ideation and suicide attempts by demographic characteristics, suicide attempts methods, COVID-19 infection, and vaccination status. Panel A Sex differences in temporal changes of suicidal ideation and suicide attempts from January 1, 2018 to November 30, 2021. Panel B Age group differences (across all age groups) in temporal changes of suicidal ideation and suicide attempts from January 1, 2018 to November 30, 2021. Panel C Age group differences (among young people under 30) in temporal changes of suicidal ideation and suicide attempts from January 1, 2018 to November 30, 2021. Panel D Ethnicity differences in temporal changes of suicidal ideation and suicide attempts from January 1, 2018 to November 30, 2021. Panel E Race differences in temporal changes of suicidal ideation and suicide attempts from January 1, 2018 to November 30, 2021. Panel F Language differences in temporal changes of suicidal ideation and suicide attempts from January 1, 2018 to November 30, 2021. *Note*. Characteristics of suicide-related visits from 2018 to 2021. Y-axis of a variable’s plot denotes the proportion of the monthly number of SI (SA) visits of the subpopulation divided by the total SI (SA) visits in the same month.

When comparing the temporal trends by demographic characteristics (Table 2 and Fig. 2A–F), we found suicidal ideation visits were consistently higher among females than males, especially during the first wave of the pandemic between March 2020 and August 2020 and the year after the pandemic between May 2021 and July 2021 (Fig. 2A). Similar patterns were observed for suicide attempt visits. Suicidal ideation visits were significantly higher for female patients during the first wave of the COVID-19 outbreak (59.9 % of total visits, *P* = 0.02) than before the COVID (57.1 % of total visits). In comparison, suicidal ideation visits for males were significantly lower during the first wave (40.1 % of total visits, *P* = 0.02) than before the COVID (42.9 % of total visits).

**Table 2:** Temporal changes in suicidal ideation and suicide attempt visits across different phases of the COVID-19 pandemic.

	Pre-COVID (2019, 6 months)	Post-pandemic (2020, 6 months)	Pre-COVID (2019, 6 months) vs post-pandemic (2020, 6 months)	Pre-vaccination COVID (12 months)	Post-vaccination COVID (12 months)	Pre-vaccination COVID (12 months) vs. post-vaccination COVID (12 months)	Pre-COVID (pre-first case)	Pre-COVID (pre-first case) vs. pre-vaccination COVID (12 months)	Pre-COVID (pre-first case) vs. post-vaccination COVID (12 months)
	01/03/2019–31/08/2019	01/03/2020–31/08/2020	*P* value	13/12/2019–12/12/2020	13/12/2020–11/30/2021	*P* value	13/12/2018–12/12/2019	*P* value	*P* value
Suicidal Ideation	*n* = *3547*	*n* = *3252*		*n* = *7647*	*n* = *9832*		*n* = *7353*		

Sex									
Male	1522 (42.9 %)	1305 (40.1)	0.02⁎	3005 (39.3 %)	3806 (38.7 %)	0.44	3084 (41.9 %)	0.002⁎⁎	<0.001⁎⁎⁎
Female	2025 (57.1 %)	1947 (59.9 %)	0.02⁎	4641 (60.7 %)	6024 (61.3 %)	0.45	4269 (58.1 %)	0.002⁎⁎	<0.001⁎⁎⁎
Unknown	0 (0.0 %)	0 (0.0 %)	–	1 (0.0 %)	2 (0.0 %)	–	0 (0.0 %)	–	–

Age (years)									
<15	358 (10.1 %)	314 (9.7 %)	0.57	929 (12.1 %)	1418 (14.4 %)	<0.001⁎⁎⁎	793 (10.8 %)	0.01⁎⁎	0.001⁎⁎
15-20	601 (16.9 %)	680 (20.9 %)	<0.001⁎⁎⁎	1666 (21.8 %)	2276 (23.1 %)	0.03	1342 (18.3 %)	<0.001⁎⁎⁎	<0.001⁎⁎⁎
21-25	436 (12.3 %)	389 (12.0 %)	0.7	915 (12.0 %)	1087 (11.1 %)	0.15	816 (11.1 %)	0.15	0.95
26–30	347 (9.8 %)	301 (9.3 %)	0.49	607 (7.9 %)	769 (7.8 %)	0.8	690 (9.4 %)	0.003⁎⁎	<0.001⁎⁎⁎
31-39	452 (12.7 %)	521 (16.0 %)	<0.001⁎⁎⁎	1019 (11.3 %)	1138 (11.6 %)	0.002⁎⁎	951 (12.9 %)	0.49	0.02⁎
40-49	405 (11.4 %)	356 (10.9 %)	0.56	807 (10.6 %)	1026 (10.4 %)	0.82	880 (12.0 %)	0.01⁎⁎	0.005⁎⁎
50-59	468 (13.2 %)	342 (10.5 %)	<0.001⁎⁎⁎	755 (9.9 %)	884 (9.0 %)	0.05	892 (12.1 %)	<0.001⁎⁎⁎	<0.001⁎⁎⁎
60+	480 (13.5 %)	349 (10.7 %)	<0.001⁎⁎⁎	949 (12.4 %)	1234 (12.6 %)	0.8	989 (13.5 %)	0.13	0.13

Race									
White	1701 (48.0 %)	1675 (51.5 %)	0.004⁎⁎	4045 (52.9 %)	5309 (54.0 %)	0.15	3650 (49.6 %)	<0.001⁎⁎⁎	<0.001⁎⁎⁎
Non-white	298 (8.4 %)	296 (9.1 %)	0.33	696 (9.1 %)	919 (9.3 %)	0.6	630 (8.6 %)	0.39	0.25
Unknown	1548 (43.6 %)	1281 (39.4 %)	<0.001⁎⁎⁎	2906 (38.0 %)	3604 (36.7 %)	0.07	3073 (41.8 %)	<0.001⁎⁎⁎	<0.001⁎⁎⁎

Ethnicity									
Hispanic	506 (14.3 %)	403 (12.4 %)	0.03⁎	908 (11.9 %)	1277 (13.0 %)	0.09	944 (12.8 %)	0.12	0.79
Non-Hispanic	1932 (54.5 %)	1964 (60.4 %)	<0.001⁎⁎⁎	4596 (60.1 %)	5992 (60.9 %)	0.27	4158 (56.5 %)	<0.001⁎⁎⁎	<0.001⁎⁎⁎
Unknown	1109 (31.3 %)	885 (27.2 %)	<0.001⁎⁎⁎	2143 (28.0 %)	2563 (26.1 %)	0.004⁎⁎	2251 (30.6 %)	<0.001⁎⁎⁎	<0.001⁎⁎⁎

Preferred language									
English	2068 (58.3 %)	2062 (63.4 %)	<0.001⁎⁎⁎	4793 (62.7 %)	6551 (66.6 %)	<0.001⁎⁎⁎	4398 (59.8 %)	<0.001⁎⁎⁎	<0.001⁎⁎⁎
Non-English	520 (14.7 %)	493 (15.2 %)	0.59	1157 (15.1 %)	1396 (14.2 %)	0.25	1053 (14.3 %)	0.25	0.84
Unknown	959 (27.0 %)	697 (21.4 %)	<0.001⁎⁎⁎	1697 (22.2 %)	1885 (19.2 %)	<0.001⁎⁎⁎	1902 (25.9 %)	<0.001⁎⁎⁎	<0.001⁎⁎⁎
Suicide Attempts	*n* = *893*	*n* = *854*		*n* = *1781*	*n* = *2309*		*n* = *1860*		

Sex									
Male	349 (39.1 %)	346 (40.5 %)	0.57	732 (41.1 %)	905 (39.2 %)	0.34	776 (41.7 %)	0.73	0.32
Female	543 (60.8 %)	508 (59.5 %)	0.61	1048 (58.8 %)	1401 (60.7 %)	0.37	1083 (58.2 %)	0.73	0.35
Unknown	1 (0.1 %)	0 (0.0 %)	–	1 (0.1 %)	3 (0.1 %)		1 (0.1 %)		

Age (years)									
<15	89 (10.0 %)	101 (11.8 %)	0.24	208 (11.7 %)	334 (14.5 %)	0.02⁎	201 (10.8 %)	0.44	0.002⁎⁎
15-20	145 (16.2 %)	146 (17.1 %)	0.68	320 (18.0 %)	457 (19.8 %)	0.23	311 (16.7 %)	0.34	0.04⁎
21-25	73 (8.2 %)	87 (10.2 %)	0.17	212 (11.9 %)	237 (10.3 %)	0.14	165 (8.9 %)	0.01⁎⁎	0.14
26-30	75 (8.4 %)	50 (5.9 %)	0.05⁎	103 (5.8 %)	146 (6.3 %)	0.52	128 (6.9 %)	0.52	0.52
31–39	113 (12.7 %)	107 (12.5 %)	0.995	214 (12.0 %)	241 (10.4 %)	0.19	229 (12.3 %)	0.82	0.19
40–49	104 (11.6 %)	114 (13.3 %)	0.32	219 (12.3 %)	261 (11.3 %)	0.53	237 (12.7 %)	0.72	0.51
50–59	127 (14.2 %)	119 (13.9 %)	0.92	226 (12.7 %)	231 (10.0 %)	0.01⁎	267 (14.4 %)	0.16	<0.001⁎⁎⁎
60+	167 (18.7 %)	130 (15.2 %)	0.06	279 (15.7 %)	402 (17.4 %)	0.29	322 (17.3 %)	0.29	0.97

Race									
White	518 (58.0 %)	493 (57.7 %)	0.95	998 (56.0 %)	1370 (59.3 %)	0.11	1077 (57.9 %)	0.37	0.37
Non-white	75 (8.4 %)	87 (10.2 %)	0.23	182 (10.2 %)	196 (8.5 %)	0.14	159 (8.5 %)	0.14	0.99
Unknown	300 (33.6 %)	274 (32.1 %)	0.54	601 (33.7 %)	743 (32.2 %)	0.55	624 (33.5 %)	0.93	0.55

Ethnicity									
Hispanic	127 (14.2 %)	127 (14.9 %)	0.75	239 (13.4 %)	305 (13.2 %)	0.91	253 (13.6 %)	0.91	0.91
Non-Hispanic	518 (58.0 %)	479 (56.1 %)	0.45	1003 (56.3 %)	1429 (61.9 %)	<0.001⁎⁎⁎	1034 (55.6 %)	0.68	<0.001⁎⁎⁎
Unknown	248 (27.8 %)	248 (29.0 %)	0.59	539 (30.3 %)	575 (24.9 %)	<0.001⁎⁎⁎	573 (30.8 %)	0.75	<0.001⁎⁎⁎

Preferred language									
English	507 (56.8 %)	507 (59.4 %)	0.29	1089 (61.1 %)	1574 (68.2 %)	<0.001⁎⁎⁎	1071 (57.6 %)	0.03⁎	<0.001⁎⁎⁎
Non-English	186 (20.8 %)	156 (18.3 %)	0.2	283 (15.9 %)	368 (15.9 %)	1	342 (18.4 %)	0.08	0.08
Unknown	200 (22.4 %)	191 (22.4 %)	1	409 (23.0 %)	367 (15.9 %)	<0.001⁎⁎⁎	447 (24.0 %)	0.47	<0.001⁎⁎⁎

Methods of suicide									
Firearms	61 (6.8 %)	73 (8.5 %)	0.21	157 (8.8 %)	120 (5.2 %)	<0.001⁎⁎⁎	109 (5.9 %)	0.001⁎⁎	0.39
Poisoning (drugs)	409 (45.8 %)	324 (37.9 %)	0.001⁎	717 (40.3 %)	934 (40.5 %)	0.93	804 (43.2 %)	0.11	0.11
Poisoning (other toxic substances)	192 (21.5 %)	239 (28.0 %)	0.002⁎	423 (23.8 %)	513 (22.2 %)	0.39	460 (24.7 %)	0.52	0.18
Other methods	231 (25.9 %)	218 (25.5 %)	0.91	484 (27.2 %)	742 (32.1 %)	0.001⁎⁎	487 (26.2 %)	0.52	<0.001⁎⁎⁎

*Note*. Characteristics of patients of suicide-related visits in the first wave of the COVID-19 outbreak and those of the corresponding period in the previous year.

⁎ *p* < 0.05.

⁎⁎ *p* < 0.01.

⁎⁎⁎ *p* < 0.001.

Suicidal ideation and suicide attempts were the highest among youth aged 15 to 20 years, followed by adults aged 31–59 and older adults aged 60 and above (Table 2, Fig. 2B). Compared to the same six-month time window before COVID-19 (01/03/2019–31/08/2019) and during COVID-19 (01/03/2020–31/08/2020), suicidal ideation increased from 16.9 % to 20.9 % (*P* < 0.001) among youth aged 15 to 20 and increased from 12.7 % to 16.0 % (*P* < 0.001, Table 2) among people aged 31 to 39. On the contrary, post-pandemic (01/03/2020–31/08/2020) suicidal ideation visits were lower than in the pre-COVID-19 period (01/03/2019–31/08/2019) for those aged 50 to 59 (decrease from 13.2 % to 10.5 %, *P* < 0.001) and for those aged over 60 (decrease from 13.5 % to 10.7 %, *P* < 0.001). Suicide attempt visits increased among youth under 15 years old between December 13, 2020, and November 30, 2021 (14.5 %), compared with the proportion one year ago (11.7 %, *P* = 0.02) and before COVID-19 (10.8 %, *P* = 0.002). On the contrary, suicide attempt visits decreased among adults aged 50–59 years old in the post-vaccination period (10.0 %) than the pre-COVID-19 period (14.4 %, *P* < 0.001). During the first year of the pandemic, suicide ideation visits were higher among people aged 31–39 than those aged 40–59 (eFigure 3). Suicide attempt visits increased among people aged 65–74 from January 2020 to November 2021 (eFigure 4).

Suicidal ideation and suicide attempt visits were consistently higher among White (Fig. 2D) and non-Hispanic (Fig. 2E) people than their counterparts. Suicide attempts among non-Hispanic patients peaked in the summer of 2020 and 2021. Compared with pre-COVID-19 periods, suicidal ideation visits increased among White and non-Hispanic people (Table 2). Suicide attempt visits among non-Hispanic people were also higher in the post-COVID-19 (both pre-and post-vaccination) period, compared with the pre-COVID-19 periods (*P* < 0.001). Suicide ideation and suicide attempt visits were higher among English-speaking patients than non-English-speaking patients (Fig. 2F).

### 3.3 Temporal differences in suicide-related visits by different suicide methods

**Fig. 3:**
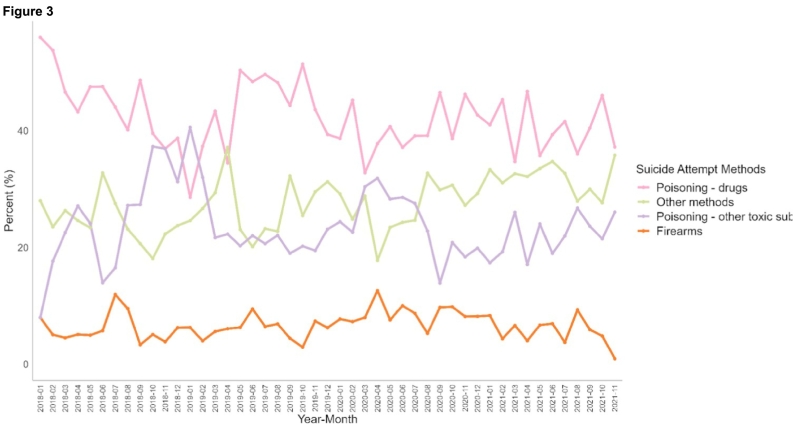
Temporal changes of suicidal ideation and suicide attempts by suicide attempts methods.

Recorded methods of suicide attempts before and after vaccination rollout were markedly different. Suicide attempts through poisoning by drugs, other methods, and poisoning by other toxic substances (e.g., nonmedicinal substances) were greater than firearm-related suicide attempts before and after the pandemic (Fig. 3). Suicide attempts by other toxic substances (e.g., nonmedicinal substances) increased in the first 6 months of the pandemic in 2020 (28.0 %) compared with that one year ago in 2019 (21.5 %, *P* = 0.002, Table 2). Firearms accounted for 8.8 % of total suicide attempts in the first year of the pandemic (13/12/2019–12/12/2020), compared to 5.2 % in the second year (13/12/2020–11/30/2021, *P* < 0.001) and 5.9 % a year before the pandemic (13/12/2018–12/12/2019, *P* < 0.001).

### 3.4 Temporal differences in suicide-related visits by COVID-19 infections and vaccinations

**Fig. 4:**
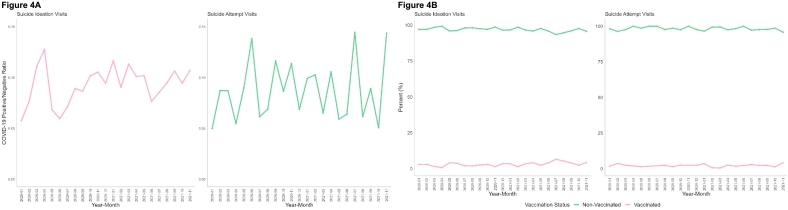
Temporal changes of suicidal ideation and suicide attempts by COVID-19 infection and vaccination status. A Suicide ideation and attempts by COVID-19 status. B Suicide ideation and attempts by vaccination status.

Suicide-related visits were consistently higher among COVID-19 negative patients than those who tested positive and among non-vaccinated patients than those who received vaccinations (Fig. 4B).

## 4 Discussion

To our knowledge, this retrospective cross-sectional study is the first to provide population-level evidence of trends in suicidal ideation or suicide attempt visits across the US before and during the COVID-19 pandemic in 2020 and 2021, compared with the previous two years (2018–2019). We used a large sample among 21,860,370 patients (30,019 suicidal ideation and 7392 suicide attempt visits) from national healthcare facility encounter data (Healthjump) that is geographically diverse and allows for detailed analyses that cannot be done with smaller samples. The study includes all ages, so trends can be examined across the lifespan, which is often impossible. Our results are also novel by considering the differences between COVID-19 testing results and COVID vaccination statuses to examine whether these factors might have had an impact on trends in suicide-related visits. Understanding the heterogeneous trends in suicide-related visits across different populations in the short- and long-term could help to identify opportunities to improve suicide prevention.

### 4.1 Importance of long-term investigation in temporal and spatial changes

Our results illuminated the need for monitoring the long-term changes in suicidal ideation and suicide attempts. Earlier data predominately reported changes in suicide deaths, stating a non-significant increase in suicide death rates (John et al., 2020; Pirkis et al., 2021), while more recent analysis started to reveal an increase in suicide death rates in the later period of the COVID-19 around December 2020 (Sakamoto et al., 2021; Tanaka and Okamoto, 2021). The longest time window in published reports on suicide-related encounters using EHR or hospital database were extracted till May 2021 in Manchester, UK (Steeg et al., 2021), or August 31, 2021, in France (Jollant et al., 2022).

We contribute to the temporal changes in non-fatal suicidal ideation and suicide attempts across different states in the US before and during COVID-19 till November 30, 2021, which is particularly valuable as previous studies are limited by geographic coverage, such as only using EHR in Massachusetts, US (Faust et al., 2021) or Manchester, UK (Appleby et al., 2021), as well as temporal coverage (e.g., only between March to May 2020).

### 4.2 Suicidal ideation and suicide attempts among populations experiencing health disparities

Our analyses are unique as we further observed significant differences during the COVID-19 pandemic by sex, age group, race, ethnicity, and preferred language. Females, youth aged 15–30, White, non-Hispanic, and English speakers had greater suicidal ideation and suicide attempt rates than their counterparts. Compared with the pre-pandemic periods, significant increases in suicidal ideation were observed among females, youth aged 15–20, 31–39, White, non-Hispanic and English speakers. Suicide attempt visits increased substantially in the later pandemic among people younger than 15, between 21 and 25 years old, non-Hispanic, and English speakers after the vaccination rollout. The disparities in suicide trends urge the need for continuous monitoring of potentially delayed suicide risks among vulnerable populations and tailoring preventative efforts to meet their social and health needs during and after the pandemics.

Our findings of the higher rates of suicide-related visits among females starting from the summer of 2020 were consistent with prior work observed in the US for suicide attempt visits in the emergency department during the early pandemic (Holland et al., 2021; Nichter et al., 2021; Ridout et al., 2021). In France, there were decreases in self-harm hospitalization in both women and men (Cousien et al., 2021; Jollant et al., 2021). Females may be more susceptible to the unintended consequences of social distancing policies and fewer social interactions while experiencing higher stressors of work-life balance and demanding childcare tasks heightened by the COVID-19 pandemic (Frank et al., 2021; Matulevicius et al., 2021). Equally concerning is the decreasing suicide-related visits among males, who may be at particularly high risk of subsequent death by suicide after self-harm but experience ongoing unmet needs of the mental health treatment (Steeg et al., 2021).

Consistent with previous findings (Cousien et al., 2021; Holland et al., 2021; Ridout et al., 2021; Steeg et al., 2021; Stephenson, 2021), we observed disproportionate growth in suicidal behaviors among patients 15–20 years old. Younger adolescents may be especially vulnerable to pandemic-related mental health illnesses from school closures, social distancing, fear of lagged school work, lack of social support, and school connectedness (Stephenson, 2021). Besides, the surge in unemployment during the first wave of COVID-19 increased mental distress among the working-age population (Berkowitz and Basu, 2021), reflected in the increase in suicide-related visits in the mid-pandemic. In line with the global literature, increases in self-harm in youth aged 10–17 were observed in Manchester, England between August 2020 and May 2021 (Steeg et al., 2021). In Ontario, Canada, increases in self-harm among youth were only observed during the second infectious wave in September 2020–January 2021 (Stewart et al., 2021), but not between the first year of the pandemic era (April 1, 2020 to June 30, 2021) compared to the 2 years before the pandemic (Ray et al., 2022). Despite the variations by the time interval, the findings of increasing suicidal ideation and attempts among youth are concerning and warrant ongoing monitoring and collective efforts among families, schools, and communities to improve resilience to pandemic disruptions (Kola et al., 2022). The decreases in suicide-related visits among patients aged 50–59 and 60 years and above may represent longer-term reluctance to seek help from health services, especially reductions in regular outpatient services during COVID-19 due to limited transportation tools to access health facilities or the intentionally delayed visits to avoid infections (Rozenfeld et al., 2020).

English speakers had greater suicide-related visits than non-English speakers since the first wave of COVID-19. On the contrary, suicidal ideation visits among non-English speakers were the highest in the first wave of the pandemic and dropped significantly since the start of vaccination. Possible explanations may be the lack of health literacy among non-English speakers while vaccination reduces the fears and uncertainties (Cohen-Cline et al., 2021; Eruchalu et al., 2021; Ingraham et al., 2021). The low suicide-related visits before and during the pandemic may also be that non-English speakers were less likely to continue accessing health facilities (Ingraham et al., 2021; Khazanchi et al., 2020).

Similar to the significant increase in drug overdose deaths in recent years (Holland et al., 2021; Khatri et al., 2021), suicide attempts by poisoning with opioids have been consistently growing since 2020, especially during the first wave (summer 2020) and post-vaccination (spring and summer 2021). The parallel increase in drug and opioid overdoses is compelling and suggests an unprecedented ‘dual pandemic’ burden, mainly due to the surges in illicitly manufactured fentanyl and stimulants (Baldwin et al., 2021). This finding may reflect heightened psychological distress and loneliness (Holland et al., 2021; Simon et al., 2020), changes in illegal drug supplies and harm-reduction services amid COVID-19 social distancing orders (Gomes et al., 2018; Lee et al., 2021). The disruptions in care among persons with opioid use disorders, particularly access to medications for addiction treatment in the first wave of COVID-19 (Alexander et al., 2020), may explain the peak of suicide attempts through poisoning by other nonmedical substances at the same time window.

This study is also among the few to document the continuous increase in suicidal ideation and suicide attempts among COVID-19 patients before and during the pandemic, even after the vaccination. People with mental illness are more susceptible to COVID-19 infections and deaths. At the same time, those who tested positive may be more likely to experience social isolation and job loss, which may increase their risks of thinking about suicide or attempting suicide (Penninx et al., 2022; Simon et al., 2020). Further studies are needed to determine the long-term impact of COVID-19 on suicidal ideation and suicide attempts and how to provide mental health service utilization among those in need during the pandemic.

### 4.3 Implications

While government social restriction policies may reduce social mobility and increase the risks of loneliness, depression, and suicide, other initiatives, including unemployment benefits (e.g., Coronavirus Aid, Relief, and Economic Security Act), providing school meals, and state-level eviction moratoriums can reduce mental distress during fall 2020. Our findings have identified the high-risk groups for intervention. Policies and programs might help to address our reported differences in sex, age groups, race, ethnicity, and methods of suicide attempts over the long term. Comprehensive and targeted suicide prevention responses are effective (Gunnell et al., 2020). For example, telehealth services and online emotional support platforms (e.g., NYC Well) shall be promoted at the individual level. At the structural level, strategies shall focus on improving access to mental health treatment and social connectedness, especially for females, younger, and non-English speakers, through offering economic support, expanding insurance coverage to telehealth, and promoting social connectedness. Societal and community-level efforts shall reinforce initiatives to mitigate misinformation, and destigmatization is strongly needed to reduce mental distress (Lai et al., 2019; Yeung et al., 2022).

### 4.4 Strengths and limitations

The strengths of our study include the use of large EHR data across the US and the ability to examine suicidal behaviors before and during COVID-19 in the longest possibility. It uniquely contributes to delineating the prolonged impact of the COVID-19 pandemic across subpopulations by sex, age groups across all lifespans, race, ethnicity, preferred languages, suicide attempt methods, and COVID-19 infections and vaccinations.

This study has limitations. First, although Healthjump covers over 55 EHR systems, data are not nationally representative, and results are not generalizable to healthcare facilities and all states. There have been incremental data in Healthjump daily. At any point, Healthjump can connect to a new data source, such as a new EHR, a provider with a new specialty, or new visit types. There will continuously be more providers being added to the dataset. Therefore, the dynamic nature of the data set provides an opportunity to offer a real-world reflection of suicidal ideation and suicide attempt visits but also prevents creating a snapshot of the data. To account for the changes, we provided the data extraction and analysis periods for each result. We also provided the total number of patients each month and adjusted percentages of suicidal ideation and suicide attempts (eTable 2, eFigure 2). Further longitudinal research is needed to clarify the causal inference of COVID-19 impact, both by the COVID-19 infection and by the disruptions associated with the pandemic, on the increases in suicidal behaviors (Czeisler et al., 2021; Penninx et al., 2022).

Second, EHRs captured patients who sought help and connected to treatment in the health care system. Yet, suicide-related visits could be influenced by the demographic characteristics of patients, changes in total visits, and accessibility of services during COVID-19. The population-level suicidal ideation and suicide attempts in the community may be higher after accounting for individuals who did not seek help or faced barriers while seeking help.

Third, like most EHR, information regarding income, education level, insurance coverage, and insurance type is not available in Healthjump. We do not have enough samples for meaningful comparisons across African-American/Black, Asian, and White.

Lastly, we used standard diagnoses and procedure codes to identify patients with positive COVID-19 test results and those who received COVID-19 vaccinations in the health systems. However, we were not able to include patients in community-based healthcare. Nevertheless, since most suicide-related visits were in office-based or ambulatory centers, we do not expect the results would be substantially different from the current analysis.

## 5 Conclusion

Surveillance of temporal trends in suicide-related visits in clinical settings among the US population is an important component of suicide prevention in the dynamic context of COVID-19. In this study, we demonstrated the longest variation in suicide-related visits in the US before and during COVID-19. We further identified the increase in suicidal behaviors among vulnerable populations by sex, age group, race, ethnicity, preferred langue, suicide methods, and COVID-19 infections and vaccination status. Such findings emphasize the need to address these vulnerable populations' social determinants of mental health experiences to effectively prepare for future public health crises. Suicide interventions and prevention in the post-pandemic era shall tailor towards special needs and risks among females, youth under 15 years old and those aged between 15 and 20 years old, White, non-Hispanic, English speakers, and people using other toxic substances for poisoning-related suicide attempts. To prevent the next storm of suicides, policymakers shall take a holistic approach and invest in social determinants of mental health disparities, including mental health treatment, access to healthy food, unemployment benefits, stable housing, and initiatives to end structural racism and discrimination.

## Funding

This research is supported by grant CORONAVIRUSHUB-D-21-00125 from the 10.13039/100000865Bill & Melinda Gates Foundation through the COVID-19 Research Accelerator Grant and the RGC Collaborative Research Fund (C7151-20G) from the University Grant Council (Hong Kong).

## Role of the funder/sponsor

The sponsor had no role in the design and conduct of the study; collection, management, analysis, and interpretation of the data; preparation, review, or approval of the manuscript; and decision to submit the manuscript for publication.

## Contributors' statement page

Dr. Xiao conceptualized and designed the study. Dr. Xiao and Mr. Junus designed the data analysis and drafted the initial manuscript. Mr. Junus and Miss Li carried out the data analysis. All authors contributed to conceptualizing the study, and reviewing and revising the manuscript.

All authors approved the final manuscript as submitted and agree to be accountable for all aspects of the work.

## CRediT authorship contribution statement

**Yunyu Xiao, Alvin Junus, Paul Yip:** Conceptualization, Methodology, Software **Yunyu Xiao, Alvin Junus:** Data curation, Writing- Original draft preparation. **Alvin Junus, Tianzi Li:** Visualization, Investigation. **Alvin Junus, Tianzi Li:** Data Analysis. **Yunyu Xiao, Paul Yip:** Supervision.: **Yunyu Xiao:** Funding acquisition.: **Yunyu Xiao, Alvin Junus, Tianzi Li, Paul Yip:** Writing- Reviewing and Editing.

## Conflict of interest

No conflicts of interest.

## Appendix A Supplementary data

Supplementary material Image 1
