# Supplementary material for: Temporal and spatial trends in suicide-related visits before and during the COVID-19 pandemic in the US, 2018–2021
Source: J Affect Disord. 2023 Mar 1;324:24–35. doi: 10.1016/j.jad.2022.12.062 (PMC9773784; doi:10.1016/j.jad.2022.12.062)
Supplement: Supplementary file 1 — Supplementary material [file mmc1.docx]

# eMethod 1 ICD and SNOMED codes of suicide attempts by methods

**Poisoning - drugs**

X60-64, T36-T50, E950.0-950.5, 960-979, E980.0-980.5, 219109002, 431307001, 61438005, 85337000

**Poisoning - other toxic substances**

X65-69, T51-65, E950.6-950.9, E951-952, E980.6-982, 242839001, 242840004, 242841000, 219122005, 219123000, 219131005, 219123000, 242836008, 242838009, 57335002, 95872005

**Hanging, strangulation, suffocation**

X70, T71, Y20, E953, E983

**Drowning**

X71, T75.1, Y21, E954, E984

**Firearms**

X73, X74, Y22, Y23, Y24, E955.0-955.4, E955.6-955.9, E985, 219334005, 219335006, 219336007, 219337003, 219338008

**Explosives**

X75, Y25

**Smoke, fire, flames**

X76, Y26, E955.5, E988.1, 219356008

**Steam, hot vapor, hot objects**

X77, Y27, E958.2, E988.2

**Sharp objects**

X78, Y28, E956, E986

**Blunt objects**

X79, Y29

**Falling, jumping**

X80, Y30, E957, E958.0, E987, E988.0, 219352005

**Crashing with moving objects**

X81, X82, Y31, Y32, E958.8, E988.5, 2193560006

**Others** (electrocution, aircraft, etc.)

X83, T75.4, 994.8, E958.3, E958.6-959, 219359001, 219361005, 243004006, 243009001

**Codes indicating undetermined intent**

Y codes excl. Y87, 960-989, E98-E99, 57335002, 61438005, 85337000, 95872005

# eTable 1 Dates of six stages of investigations from pre- COVID-19 to post COVID-19 period

| **Dates** | **Stages** |
| --- | --- |
| 01/01/2018 – 11/12/2019 | Pre-COVID-19, Pre First Case |
| 12/12/2019 – 29/02/2020 | Pre-COVID-19, Pre-Pandemic |
| 01/03/2020 – 31/08/2020 | Post-COVID-19, First Wave |
| 01/09/2020 – 12/12/2020 | Post-COVID-19, Post-First Wave, Pre-Vaccination |
| 13/12/2020 – 26/07/2021 | Post-COVID-19, Post-Vaccination, Pre-Delta |
| 27/07/2021 – 30/11/2021 | Post-COVID-19, Delta wave |

# eTable 2 Frequencies and percentages of monthly suicidal ideation and suicide attempts from January 2018 to November 2021

| **Month-Year** | **SI (frequency)** | **SA (frequency)** | **SI (per 1,000 providers)** | **SA (per 1,000 providers)** | **Patient** | **SI (%)** | **SA (%)** |
| --- | --- | --- | --- | --- | --- | --- | --- |
| January 2018 | 495 | 100 | 745 | 151 | 2,053,631 | 0.02% | 0.00% |
| February 2018 | 400 | 119 | 603 | 179 | 1,955,993 | 0.02% | 0.01% |
| March 2018 | 466 | 133 | 700 | 200 | 2,020,006 | 0.02% | 0.01% |
| April 2018 | 472 | 118 | 700 | 175 | 2,001,249 | 0.02% | 0.01% |
| May 2018 | 425 | 141 | 633 | 210 | 2,060,180 | 0.02% | 0.01% |
| June 2018 | 371 | 122 | 550 | 181 | 1,962,747 | 0.02% | 0.01% |
| July 2018 | 387 | 109 | 568 | 160 | 1,969,591 | 0.02% | 0.01% |
| August 2018 | 532 | 147 | 781 | 216 | 2,171,977 | 0.02% | 0.01% |
| September 2018 | 547 | 150 | 801 | 220 | 1,992,420 | 0.03% | 0.01% |
| October 2018 | 618 | 177 | 896 | 257 | 2,412,492 | 0.03% | 0.01% |
| November 2018 | 540 | 130 | 785 | 189 | 2,170,978 | 0.02% | 0.01% |
| December 2018 | 502 | 160 | 730 | 233 | 2,010,311 | 0.02% | 0.01% |
| January 2019 | 586 | 175 | 844 | 252 | 2,335,830 | 0.03% | 0.01% |
| February 2019 | 535 | 150 | 769 | 216 | 2,194,730 | 0.02% | 0.01% |
| March 2019 | 620 | 143 | 893 | 206 | 2,321,076 | 0.03% | 0.01% |
| April 2019 | 617 | 148 | 883 | 212 | 2,348,617 | 0.03% | 0.01% |
| May 2019 | 598 | 143 | 854 | 204 | 2,360,470 | 0.03% | 0.01% |
| June 2019 | 510 | 159 | 729 | 227 | 2,176,663 | 0.02% | 0.01% |
| July 2019 | 571 | 155 | 815 | 221 | 2,317,226 | 0.02% | 0.01% |
| August 2019 | 631 | 145 | 896 | 206 | 2,424,659 | 0.03% | 0.01% |
| September 2019 | 721 | 158 | 1026 | 225 | 2,503,710 | 0.03% | 0.01% |
| October 2019 | 762 | 173 | 1076 | 244 | 2,885,347 | 0.03% | 0.01% |
| November 2019 | 680 | 149 | 958 | 210 | 2,513,233 | 0.03% | 0.01% |
| December 2019 | 640 | 160 | 900 | 225 | 2,508,812 | 0.03% | 0.01% |
| January 2020 | 734 | 168 | 1029 | 236 | 2,784,490 | 0.03% | 0.01% |
| February 2020 | 733 | 137 | 1025 | 192 | 2,630,046 | 0.03% | 0.01% |
| March 2020 | 511 | 125 | 714 | 175 | 2,438,603 | 0.02% | 0.01% |
| April 2020 | 389 | 135 | 543 | 189 | 1,875,857 | 0.02% | 0.01% |
| May 2020 | 516 | 145 | 719 | 202 | 2,136,824 | 0.02% | 0.01% |
| June 2020 | 586 | 140 | 822 | 196 | 2,620,827 | 0.02% | 0.01% |
| July 2020 | 603 | 138 | 854 | 195 | 2,707,612 | 0.02% | 0.01% |
| August 2020 | 647 | 171 | 915 | 242 | 2,696,054 | 0.02% | 0.01% |
| September 2020 | 790 | 144 | 1110 | 202 | 2,932,018 | 0.03% | 0.00% |
| October 2020 | 844 | 163 | 1179 | 228 | 3,171,049 | 0.03% | 0.01% |
| November 2020 | 640 | 147 | 899 | 206 | 2,852,913 | 0.02% | 0.01% |
| December 2020 | 684 | 171 | 958 | 239 | 2,923,824 | 0.02% | 0.01% |
| January 2021 | 738 | 144 | 1041 | 203 | 2,928,841 | 0.03% | 0.00% |
| February 2021 | 783 | 161 | 1106 | 227 | 2,806,728 | 0.03% | 0.01% |
| March 2021 | 954 | 196 | 1355 | 278 | 3,325,551 | 0.03% | 0.01% |
| April 2021 | 968 | 199 | 1369 | 281 | 3,202,611 | 0.03% | 0.01% |
| May 2021 | 779 | 179 | 1100 | 253 | 2,927,098 | 0.03% | 0.01% |
| June 2021 | 773 | 216 | 1100 | 307 | 3,075,933 | 0.03% | 0.01% |
| July 2021 | 761 | 214 | 1086 | 305 | 3,001,992 | 0.03% | 0.01% |
| August 2021 | 751 | 172 | 1088 | 249 | 3,255,252 | 0.02% | 0.01% |
| September 2021 | 852 | 220 | 1249 | 323 | 3,188,766 | 0.03% | 0.01% |
| October 2021 | 846 | 228 | 1239 | 334 | 3,191,855 | 0.03% | 0.01% |
| November 2021 | 911 | 215 | 1350 | 319 | 3,049,256 | 0.03% | 0.01% |
| Percentage of Changes | 84.0% | 115.0% | 81.2% | 111.3% |  | 23.9% | 44.8% |
| Ratio of frequencies | 1.8 | 2.2 | 1.8 | 2.1 |  | 1.2 | 1.4 |

Note: SI=suicidal ideation; SA=suicide attempts. Percentages are calculated using the frequency of SI/SA divided by the total number of patients in the same month and year.

# eFigure 1 Spatial variations in the number of health providers with suicidal ideation and suicide attempt visits from 2018 to 2021

# eFigure 2 Frequencies and percentages of suicidal ideation and suicide attempts between January 2018 and November 2021

# eFigure 3 Age group differences (among adults between 31 and 59) in temporal changes of suicidal ideation and suicide attempts from January 1, 2018, to November 30, 2021

# eFigure 4 Age group differences (among older adults above 60 years old) in temporal changes of suicidal ideation and suicide attempts from January 1, 2018, to November 30, 2021.
